# Reducing Clinical Trial Monitoring Resources and Costs With Remote Monitoring: Retrospective Study Comparing On-Site Versus Hybrid Monitoring

**DOI:** 10.2196/42175

**Published:** 2023-06-27

**Authors:** Zhiying Fu, Xiaohong Liu, Shuhua Zhao, Yannan Yuan, Min Jiang

**Affiliations:** 1 Peking University Cancer Hospital & Institute Beijing China

**Keywords:** clinical trial, management, on-site monitoring, hybrid monitoring model, remote monitoring, hybrid, monitoring, cost, economic, trial monitoring, research quality, scientific research, trials methodology, trial management, research management

## Abstract

**Background:**

Clinical research associates (CRAs) monitor the progress of a trial, verify the data collected, and ensure that the trial is carried out and reported in accordance with the trial protocol, standard operating procedures, and relevant laws and regulations. In response to monitoring challenges during the COVID-19 pandemic, Peking University Cancer Hospital launched a remote monitoring system and established a monitoring model, combining on-site and remote monitoring of clinical trials. Considering the increasing digitization of clinical trials, it is important to determine the optimal monitoring model for the general benefit of centers conducting clinical trials worldwide.

**Objective:**

We sought to summarize our practical experience of a hybrid model of remote and on-site monitoring of clinical trials and provide guidance for clinical trial monitoring management.

**Methods:**

We evaluated 201 trials conducted by our hospital that used on-site monitoring alone or a hybrid monitoring model, of which 91 trials used on-site monitoring alone (arm A) and 110 used a hybrid model of remote and on-site monitoring (arm B). We reviewed trial monitoring reports from June 20, 2021, to June 20, 2022, and used a customized questionnaire to collect and compare the following information: monitoring cost of trials in the 2 models as a sum of the CRAs’ transportation (eg, taxi fare and air fare), accommodation, and meal costs; differences in monitoring frequency; the number of monitored documents; and monitoring duration.

**Results:**

From June 20, 2021, to June 20, 2022, a total of 320 CRAs representing 201 sponsors used the remote monitoring system for source data review and the verification of data from 3299 patients in 320 trials. Arm A trials were monitored 728 times and arm B trials were monitored 849 times. The hybrid model in arm B had 52.9% (449/849) remote visits and 48.1% (409/849) on-site visits. The number of patients’ visits that could be reviewed in the hybrid monitoring model increased by 34% (4.70/13.80; *P*=.004) compared with that in the traditional model, whereas the duration of monitoring decreased by 13.8% (3.96/28.61; *P*=.03) and the total cost of monitoring decreased by 46.2% (CNY ¥188.74/408.80; *P*<.001). These differences were shown by nonparametric testing to be statistically significant (*P*<.05).

**Conclusions:**

The hybrid monitoring model can ensure timely detection of monitoring issues, improve monitoring efficiency, and reduce the cost of clinical trials and should therefore be applied more broadly in future clinical studies.

## Introduction

Monitoring is an important means of ensuring the smooth implementation and quality of clinical trials [1]. A clinical research associate (CRA) is an individual who possesses specialized knowledge about clinical trials and has been appointed by the trial sponsor. CRAs monitor the progress of a trial; verify the data collected; and ensure that the trial is carried out, documented, and reported in accordance with the trial protocol, standard operating procedures, and relevant laws and regulations. The standard monitoring model involves regular site visits for on-site monitoring during trial conduct.

The use of information technology to improve efficiency and reduce the cost of clinical trials is of increasing interest among all clinical trial stakeholders. In 2020, the COVID-19 pandemic prompted an urgent demand for remote monitoring of clinical trials [2]. Guidelines for conducting clinical trials during the COVID-19 pandemic were subsequently issued by the US Food and Drug Administration [3], European Medicines Agency [4], and Association Contract Research Organization [5]. A remote approach to monitoring was proposed to protect patients and facilitate the continuation of trials while maintaining Good Clinical Practice (GCP) standards for trial execution [6]. GCP (2020) in China [7] indicates that different monitoring methods can improve the efficiency and effectiveness of trial monitoring. The National Medical Products Administration (NMPA) Center for Drug Evaluation (CDE) states in the *Guidelines for the Management of Drug Clinical Trials during the Epidemic Period of New Coronary Pneumonia (Interim)* [8] that “digital technology combining centralized monitoring and remote monitoring can be used to carry out drug clinical trials during the epidemic period.” The proposed remote source data verification (SDV) strategy includes access to files through the patients’ electronic health records and clinical database, with web-based access technology assisting the monitoring effort [9].

Remote monitoring can be implemented in a variety of ways, but it is largely dependent on the infrastructure and investment of the field center [10]. Relying on long-term informatization, Peking University Cancer Hospital launched a remote monitoring system in February 2020 so that monitoring is not affected by factors such as pandemic restrictions, location, or time and thus maximizes the health and safety of individuals participating in clinical trials as well as trial quality [11]. Remote monitoring has gradually become a necessary tool for monitoring work to continue as the pandemic progressed [12], promoting a change in the clinical trial monitoring model and the evolution of a new monitoring model, combining on-site and remote monitoring. We sought to summarize the practical experience of using a hybrid model of remote monitoring at our hospital and compare the efficiency of routine on-site monitoring with the hybrid monitoring model, thus providing a useful reference for future clinical trial monitoring management.

## Methods

### Ethics Approval

The study was approved by the Ethics Review Committee of Peking University Cancer Hospital (2020YW135), which waived the requirement for patients to provide informed consent because all had already provided written informed consent for their medical data to be analyzed and published in an anonymized format for medical research purposes. All CRAs of the included trials consented voluntarily to answer the questionnaire without compensation.

### Study Hypothesis

The hybrid mode of on-site monitoring combined with remote monitoring can improve monitoring efficiency and reduce the cost of clinical trials compared to that of traditional on-site monitoring alone.

### Study Design

We included trials that used on-site monitoring alone (arm A) or a hybrid monitoring model (arm B) at our hospital from June 20, 2021, to June 20, 2022. Using data from monitoring reports and a tailored questionnaire survey on the monitoring cost of trials, differences in the monitoring frequency, average workload, cost, and monitoring duration were compared between the routine on-site monitoring model and the hybrid monitoring model.

In the hybrid mode of the monitoring, the following routine monitoring activities were remotely conducted: source document review including informed consent process documentation, medical notes, laboratory results, investigational product (IP) storage and dispensing records, and SDV. Activities that could not be performed remotely were performed during on-site visits, including original signed informed consent form review, IP accountability, and face-to-face meetings with the principal investigator.

### Data Sources and Collection

Using background data from our remote monitoring system, information related to the use of remote monitoring including the number of monitors, monitoring frequency, the number of trials, and the number of included patients were collected from the period of June 20, 2021, to June 20, 2022.

On June 20, 2022, we released trial recruitment information in the CRA instant message application group (WeChat), and the recruitment criteria were (1) phase I to III drug clinical trials, (2) trials in either the enrollment period or the treatment follow-up period, and (3) trials that used on-site monitoring alone or a hybrid monitoring model. The recruitment lasted for 3 days.

Basic information on the included trials was collected from our hospital clinical trial management system, including trial phase, the type of trial, blinding status, IP category, single treatment or combination medication, enrollment period or treatment follow-up period, and the number of patients monitored.

During trial conduct, the CRA checks clinical trial data and procedures through remote or on-site monitoring and summarizes the findings (including data inconsistencies and protocol deviations) in a monitoring report. From the monitoring reports generated for each included trial between June 20, 2021, and June 20, 2022, we extracted data on the number of on-site and remote monitoring visits and the workload and time spent on monitoring in each monitoring model within 1 year, including the following parameters: (1) the number of visits and time taken to review all patients; (2) the number of adverse events and concomitant medication recorded in the original records and the duration of review; (3) the number of case report form (CRF) pages verified and the duration of review; and (4) total monitoring duration.

The monitoring cost of each included trial was collected using a custom questionnaire (Multimedia Appendix 1) from June 20, 2022, to June 27, 2022. We calculated the total monitoring cost in the 2 models as a sum of the CRAs’ transportation (eg, taxi fare and air fare), accommodation, and meal costs.

### Statistical Methods

SPSS (version 26.0; IBM Corp) software was used to perform descriptive analysis and normal distribution tests on the data collected in the included trials. The differences in basic characteristics were assessed for significance using the chi-square test. Where data were not normally distributed, a nonparametric test was used to compare differences in monitoring frequency, average workload, duration, and monitoring costs between the on-site and hybrid monitoring models.

## Results

### Design and Implementation of Remote Monitoring at Our Hospital

Our hospital constructed a hospital-wide “Intelligent Platform for Clinical Trial Data” in 2018 [13]. This data processing and application platform can automatically collect, merge, standardize, and structure all diagnosis and treatment data, forming a personal health standardized record. In 2019, our hospital developed a remote monitoring system based on this platform. The remote monitoring system integrates all data on patient visits according to regulatory requirements and presents an overview of the patient with respect to visit parameters including examinations, medical records, diagnoses, and treatments without the hospital information system. Electronic health record source data collected per patient during a clinical trial were obtained under GCP regulations [7], and Health Insurance Portability and Accountability Act principles were referenced regarding the use and protection of patients’ personal information (Medical Electronic Exchange Act published by the US Department of Health) [14]. Sensitive personal information such as name, ID number, telephone number, and address was anonymized. An account watermark was added to the panoramic page of patients to verify the protection of patients’ clinical trial data.

**Figure 1:**
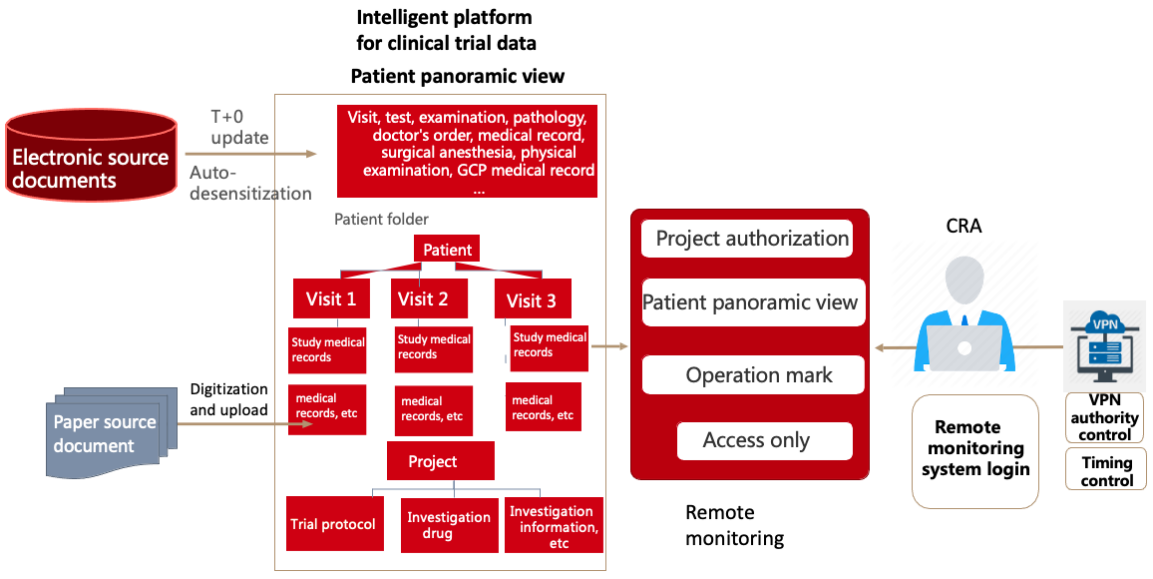
Overall implementation scheme of the remote monitoring system in our hospital. CRA: clinical research associate; GCP: Good Clinical Practice; VPN: virtual private network.

**Figure 2:**
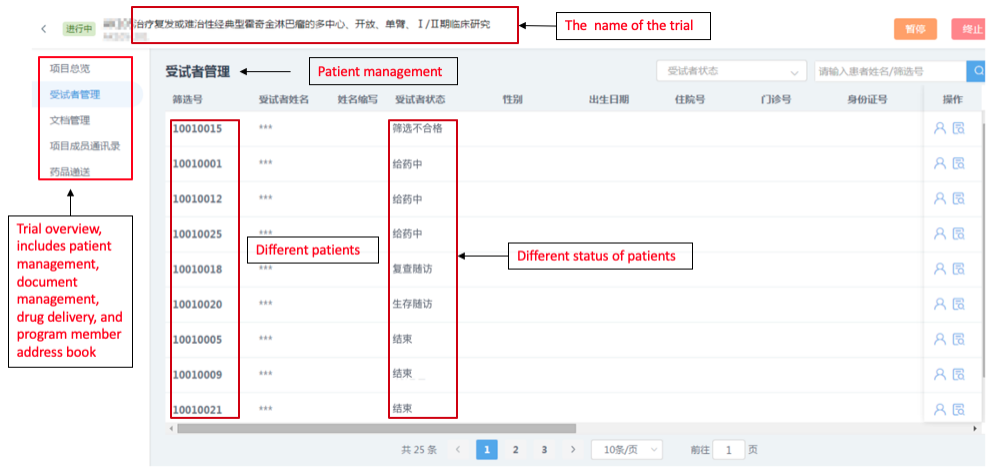
Screenshot of the panoramic view of a clinical trial in the remote monitoring system.

**Figure 3:**
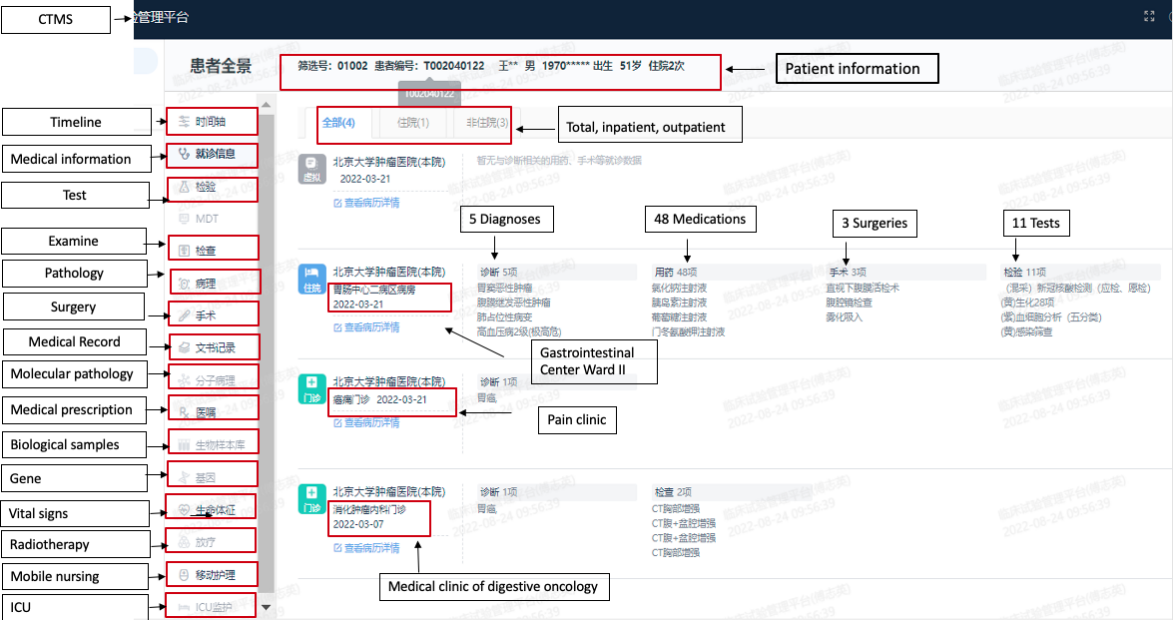
Screenshot of the panoramic view of a clinical trial participant in the remote monitoring system. CTMS: clinical trial management system; ICU: intensive care unit.

CRAs were able to access the remote monitoring system with permission to view all data from anonymized patients, including progress notes on outpatient and inpatient treatment, medical records, examinations, and tests. In the clinical trials, nonelectronic patient data and investigators’ files were anonymized and uploaded to the remote monitoring system to facilitate remote monitoring of all clinical trial data. The overall implementation scheme of the remote monitoring system is shown in Figure 1. The remote monitoring trial and patient review interfaces are shown in Figures 2 and 3.

### System Application

**Figure 4:**
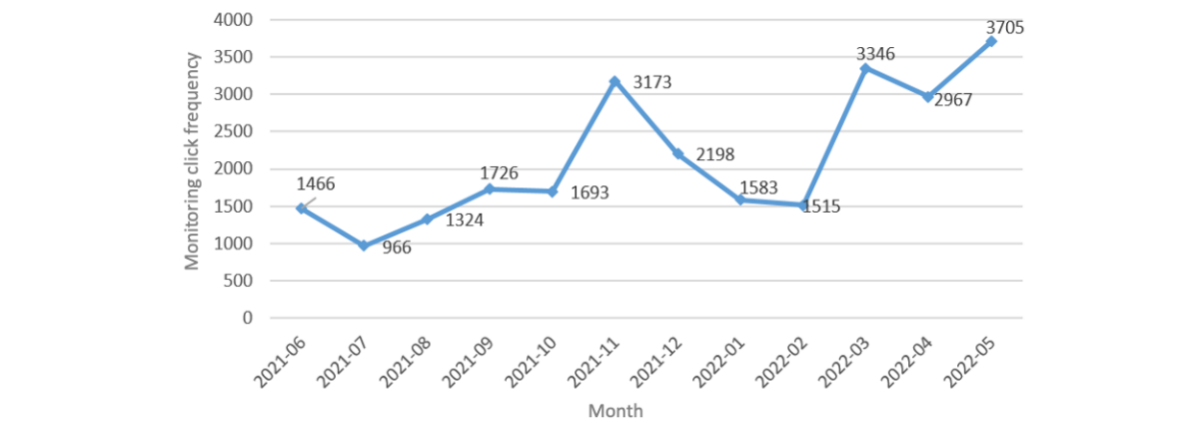
Monthly frequency trend chart of remote monitoring in our hospital.

Our hospital’s remote monitoring system was launched on February 20, 2020. From June 20, 2021, to June 20, 2022, background data from the remote monitoring system showed that 320 CRAs at our hospital used the system to conduct remote review and verification in 320 trials involving 3299 patients. The total monitoring click frequency was 27,837 times (Figure 4). See Figure 3 for details of the monitoring system home screen.

### Comparison Between the On-Site and Hybrid Monitoring Models

We included a total of 201 trials in our analysis, of which 91 trials used on-site monitoring alone (arm A) and 110 used the hybrid model of remote monitoring and on-site monitoring (arm B, which are included in the 320 trials mentioned). The chi-square test showed that there was no significant difference in basic characteristics between trials using routine on-site monitoring and those using hybrid monitoring (all *P*>.05; Table 1). Of note, monitoring tasks were the same for both models.

A total of 201 questionnaires were collected and analyzed, and the results showed that 728 on-site monitoring visits were performed in arm A. In arm B, a total of 849 monitoring visits were performed using on-site monitoring and remote monitoring approaches. Of these visits, 52.9% (449/849) were remote visits and 48.1% (409/849) were on-site visits. In arm A, data from 6625 patient visits and 31,675 adverse events and concurrent medications were monitored within the 1-year study period, and 105,909 pages of CRFs were reviewed. In arm B, data from 11,716 patient visits and 39,894 adverse events and concurrent medications were monitored, and 134,643 pages of CRFs were reviewed.

**Table 1:** Basic characteristics of trials adopting routine on-site monitoring and hybrid monitoring.

Category		Routine on-site monitoring (n=91)	Hybrid monitoring (n=110)	*P* value
**Trial phase** **, n (%)**				
	Phase I	18 (19.8)	27 (24.5)	.14
	Phase II	24 (26.4)	38 (34.5)	
	Phase III	49 (53.8)	45 (40.9)	
**Blinding method** **, n (%)**				
	Yes	25 (27.5)	18 (16.4)	.06
	No	66 (72.5)	92 (83.6)	
**Medication category** **, n (%)**				
	Biologics	66 (72.5)	78 (70.9)	.80
	Chemicals	25 (27.5)	32 (29.1)	
**Administration** **, n (%)**				
	Single medication	43 (47.3)	53 (48.2)	.90
	Combination	48 (52.7)	57 (51.8)	
**Nature of the trial** **, n (%)**				
	Local	61 (67)	81 (73.6)	.51
	Multinational	30 (33)	29 (26.4)	
**Trial stage** **, n (%)**				
	Enrollment period	45 (49.5)	67 (60.9)	.10
	Follow-up period	46 (50.5)	43 (29.1)	
Number of patients, mean (SD)		9.13 (12.739)	9.87 (11.669)	.44

On average, the number of all visits in which patients could complete a review per monitoring visit increased by 34% (4.70/13.80; *P*=.004), the length of monitoring decreased by 13.8% (3.96/28.61; *P*=.03), and the monitoring cost decreased by 46.2% (CNY ¥188.74/408.80; CNY ¥7.15=US $1; *P*<.001) in the hybrid monitoring model compared with that in the on-site monitoring model (Table 2).

**Table 2:** Comparison of the efficiency of routine on-site monitoring and hybrid monitoring model.

Issue category per monitoring visit	Routine on-site monitoring, mean (SD)	Hybrid monitoring model, mean (SD)	*P* value
Number of patients	9.10 (11.166)	13.80 (19.967)	.004
Time consumption (hours)	11.63 (8.969)	10.73 (8.883)	.13
Number of AE^a^ and concomitant medications	43.51 (52.096)	46.99 (55.718)	.45
Time consumption for AE and concomitant medications (hours)	7.23 (7.384)	6.46 (6.436)	.56
Number of CRF^b^ pages	145.48 (135.356)	158.59 (151.562)	.66
Time consumption for reviewing CRF pages (hours)	10.27 (11.465)	8.77 (6.484)	.91
Total monitoring time (hours)	28.61 (20.719)	24.650 (20.950)	.03
Total monitoring cost (CNY ¥; CNY ¥7.15=US $1)	408.80 (688.131)	220.06 (337.367)	<.001

^a^AE: adverse events.

^b^CRF: case report form.

## Discussion

### Principal Findings

Remote monitoring of clinical trials is an innovative applications of medical big data technology, promoting the implementation of patient-centered digital technology for clinical trials. At present, domestic and international investigators are actively exploring the construction and standardization of remote monitoring systems [12,15-17]. However, few reports to date have examined the effectiveness of remote monitoring. Uren et al [18] demonstrated overall trial cost savings using a hybrid monitoring model in a phase III clinical study with a 2:1 ratio of remote monitoring to on-site monitoring. However, a major limitation of this study was the inclusion of only 4 patients. Our study evaluated data from a total of 201 trials to determine the effectiveness of hybrid monitoring versus standard on-site monitoring.

At present, remote monitoring practices domestically and overseas use one of three main approaches: (1) the Florence remote monitoring system in the United States links to the original hospital data, allowing the collection and acquisition of electronic data in clinical trials to facilitate remote monitoring [19]; (2) a solution for uploading multicenter anonymized data to a single platform for remote review [20]; and (3) scanning of all trial documents for manual anonymization before remote review [21]. Our hospital has pioneered the connection model, whereby real-time transmission of anonymized medical data in electronic source files and scans of paper document are used to form a complete data chain mode. Paper trial documents such as the informed consent form, drug record sheet, and laboratory sample collection record sheet are scanned using a high-speed scanner, automatically anonymized, and uploaded to the remote monitoring system, thus providing a comprehensive and coherent data source for remote monitoring.

Compared with on-site monitoring alone, the hybrid monitoring model has the following advantages: (1) remote monitoring can use visual data to more quickly and frequently evaluate data and information, facilitate queries, and identify focus areas for subsequent on-site monitoring; (2) the efficiency of on-site monitoring can be substantially improved by reducing the duration and cost of monitoring; (3) using remote monitoring, protocol compliance and safety can be assessed more quickly, early safety signals or trends can be identified, and real-time monitoring can be performed to ensure the safety of patients and improve the quality of the trial; and (4) investigators can be alerted to safety and protocol compliance issues in a timely manner, thus minimizing the risk of recurrence.

We conducted in-depth interviews with 16 CRAs previously to understand how the remote monitoring and on-site monitoring cooperate and to give propositions for further improvement [22]. SDV is a time-consuming part of the monitoring tasks, which is more suitable for the hybrid mode of on-site and remote monitoring. The application of a hybrid model requires trial-related data to be electronically generated as much as possible. Documents or data that are generated on paper should be scanned and uploaded to the data capture system so that remote monitoring can be performed. Only a few tasks such as IP accountability and investigator site file review must be done during an on-site visit. By prespecifying project-specific parameters such as patient visit schedule and laboratory reference range values, the data management system can automatically check electronic data and raise queries in the system. Hence, site staff and monitors can verify data based on the raised queries. The more electronic data that are available for web-based review, the more suitable the study is for remote monitoring (and the more efficient it is in terms of on-site monitoring time as most tasks can be done in-house, reducing time spent on-site and saving on travel costs).

International remote monitoring regulations and applications are at an early stage, with further standardization and clarification of the process required. In November 2021, the effective implementation of the Personal Information Protection Law of the People’s Republic of China [23] provided the legal basis for the protection and management of personal information of patients during remote monitoring. Issued by the NMPA CDE on August 9, 2022, the *Technical Guidelines for the Implementation of Patient-centered Clinical Trials (Draft for Comments)* [24] proposed that the primary concern for remote monitoring is the protection of the patient’s personal information and the safety of the data. To facilitate compliant use of clinical medical data, the remote monitoring platform should perform system verification, ensure the use of security measures such as data encryption or the anonymization of source data, and specify the access rights and access range of each system. For specific implementation and management, and to meet basic GCP requirements, remote monitoring can refer to relevant documents or industry consensus from the US Food and Drug Administration, European Medicines Agency, and medical information industry organizations. Investigator sites should establish robust operational specifications and quality management systems for remote monitoring; prevent and control various risks in clinical trials, such as untimely data transmission, errors, and deficiencies; and provide efficient and convenient communication and solution channels when issues arise.

Prior to the COVID-19 pandemic, progress in using noncentralized clinical trial models and work related to remote data were limited [25]. However, telemedicine has rapidly gained popularity since the pandemic, along with technologies that support telemedicine models [26]. In meeting compliance and data security requirements, the remote monitoring system addresses historical limitations of clinical trial monitoring by providing direct access to source documents in different environments such as audits and remote authority inspections. Furthermore, the use of remote systems promotes the formation of a safe and open clinical trial data network alliance, paperless clinical trials, and the development and use of emerging clinical models such as decentralized clinical trials.

Our study had a number of limitations, including its retrospective design with a risk of selection and information bias, and the clinical trials were not randomly assigned to the arms, so could there be confounding factors contributing to the differences. Only 201 trials were included in this study, and the sample size will be expanded in further research in the future. With the continuous application of the hybrid monitoring model, prospective studies may be used to evaluate the effectiveness and efficiency of this monitoring approach.

### Conclusion

The hybrid model combining remote monitoring with on-site monitoring of clinical trials can reduce monitoring frequency, improve monitoring efficiency, save costs, improve monitoring quality, and facilitate data use that is more in line with the GCP guidelines from the International Council for Harmonisation. The implementation of the hybrid monitoring model is relatively mature at present, and continuous advances are expected. Medical institutions may interact with sponsors and regulatory authorities of the NMPA to establish an intelligent implementation and management plan for the entire lifecycle of clinical trials, thus facilitating clinical studies that are truly patient centered.

## Appendix

Multimedia Appendix 1 Monitoring model questionnaires (arm A and arm B).

## Abbreviations

CDE: Center for Drug Evaluation
CRA: clinical research associate
CRF: case report form
GCP: Good Clinical Practice
IP: investigational product
NMPA: National Medical Products Administration
SDV: source data verification

## Data Availability

The data sets used and/or analyzed in this study can be made available by the corresponding author upon reasonable request.
