# Supplementary material for: Reducing Clinical Trial Monitoring Resources and Costs With Remote Monitoring: Retrospective Study Comparing On-Site Versus Hybrid Monitoring
Source: J Med Internet Res. 2023 Jun 27;25:e42175. doi: 10.2196/42175 (PMC10337412; doi:10.2196/42175)
Supplement: Multimedia Appendix 1 [file jmir_v25i1e42175_app1.docx]

| \| Monitoring Model Questionnaire for Clinical Research Associate(Arm A)  This survey is conducted anonymously and the information obtained will be used only for the purpose of monitoring services. You can voluntarily decide whether or not to participate in this survey.  According to your work situation in the past 1 year, please fill the best answer in this blank. Your answers will provide useful references for improving your working conditions in the future. Please answer each question carefully. \| \| --- \| \| 1. Trial number ____ \| \| 2. Trial name ____ \| \| 3. What stage is the trial at the present stage.____ \| \| 4. Number of subjects per monitoring review in on-site monitoring model within a year. ____  5.Time consumption per reviewing visits per monitoring in on-site monitoring model within a year. ____  6.The number of on-site monitoring model conducted within a year in on-site monitoring model within a year. ____ \| \| 7. Number of Average Adverse event (AE) and Concomitant Medications(CM) per monitoring review in on-site monitoring model. ____ \| \| 8. Time consumption AE and CM per monitoring in on-site monitoring model within a year. ____ \| \| 9. Number of Case Report Form (CRF) pages per monitoring review in on-site monitoring model within a year. ____ \| \| 10. Tme consumption reviewing CRF pages per monitoring in on-site monitoring model within a year. ____ \| \| 11. Total monitoring time per monitoring in on-site monitoring model within a year. ____ \| \| 12. The total monitoring cost per monitoring in on-site monitoring model within a year, as a sum of the monitors’ transportation (e.g., taxi fare and air fare), accommodation, and meal costs. ____ \|     Monitoring Model Questionnaire for Clinical Research Associate(Arm B)  This survey is conducted anonymously and the information obtained will be used only for the purpose of monitoring services. You can voluntarily decide whether or not to participate in this survey.  According to your work situation in the past 1 year, please fill the best answer in this blank. Your answers will provide useful references for improving your working conditions in the future. Please answer each question carefully. |
| --- | --- | --- | --- | --- | --- | --- | --- | --- | --- | --- | --- |
| 1. Trial number ____ |
| 2. Trial name ____ |
| 3. What stage is the trial at the present stage____ |
| 4. Number of subjects per monitoring review in a monitoring model combining on-site and remote monitoring (hybrid monitoring model) within a year. ____  5.Time consumption per reviewing visits per monitoring in hybrid monitoring model within a year. ____  6.The number of hybrid monitoring model conducted within a year in hybrid monitoring model within a year. ____ |
| 7. Number of Average Average Adverse event (AE) and Concomitant Medications(CM) per monitoring review. ____ |
| 8. Time consumption AE and CM per monitoring in hybrid monitoring model within a year. ____ |
| 9. Number of Case Report Form (CRF) pages per monitoring review in hybrid monitoring model within a year. ____ |
| 10. Tme consumption reviewing CRF pages per monitoring in hybrid monitoring model within a year. ____ |
| 11. Total monitoring time per monitoring in hybrid monitoring model within a year. ____ |
| 12. The total monitoring cost per monitoring in hybrid monitoring model within a year, as a sum of the monitors’ transportation (e.g., taxi fare and air fare), accommodation, and meal costs. ____ |
